# Are we ready for building transition programs for heart transplant recipients in Japan? – Knowing the unique background is the first step for discussion

**DOI:** 10.3389/fped.2022.935167

**Published:** 2022-11-04

**Authors:** Tomoko S. Kato, Harumi Gomi, Yoshiyasu Aizawa, Akio Kawamura, Howard J. Eisen, Sharon A. Hunt, Takamitsu Inoue

**Affiliations:** ^1^ Department of Cardiology, International University of Health and Welfare School of Medicine, Narita, Chiba, Japan; ^2^ Office of Medical Education and Center for Infectious Diseases, International University of Health and Welfare School of Medicine, Narita, Chiba, Japan; ^3^ Department of Medicine, Division of Cardiology, Penn State Heart and Vascular Institute, Harrisburg, PA, United States; ^4^ Department of Medicine, Division of Cardiology, Stanford University, Stanford, CA, United States; ^5^ Department of Renal and Urological Surgery, International University of Health and Welfare School of Medicine, Narita, Chiba, Japan

**Keywords:** healthcare transition, heart transplant, sociocultural factors, Japan, paternalism

## Introduction

Although Japan is a developed country from an economic, educational, and academic viewpoint, certain areas in medicine, such as transplantation and the transition of patients with childhood-onset chronic diseases from pediatric to adult healthcare systems, have fallen far behind the United States (US) or European countries. Especially concerning solid organ transplantation, heart transplantation (HTx) in Japan has a tragic history that hinders active discussion in this field. In this article, we aim to highlight the underlying issues surrounding both HTx and transition medicine in Japan as a preliminary proposal to initiate a constructive discussion to encourage future perspectives.

Blum RW et al. defined transition as the “purposeful, planned movement of adolescents and young adults with chronic physical and medical conditions from child-centered to adult-oriented health care systems” (1). The goal of a transition is to provide uninterrupted, coordinated, developmentally appropriate, psychosocially sound, and comprehensive healthcare for adolescents and young adults (1).

Thanks to advances in surgical techniques and improvements in immunosuppressants, more pediatric solid-organ transplant recipients are surviving to adulthood (2, 3). In general, the period of transition is associated with a risk of non-adherence to medical treatment or lapses in care (4, 5). Such puberty-related non-compliance issues are impactful for transplant recipients, because they directly lead to life-threatening events or graft failure. Transplant recipients tend to be psychosocially immature, and one of the reasons for this is less exposure to their peers during the pre-transplant period (6). Parents' experience with the fear that they may lose their child to a life-threatening disease may also influence the difficulties in shifting responsibility for care from the parents to the patients themselves. Even so, adolescent and young adult transplant recipients are expected to have the capability to manage their complex medication, independently discuss their condition with their treatment team, and schedule their appointments (7), which can be considered a goal for transition in transplant recipients. In recent years, establishing a successful transition from pediatric- to adult-focused transplant programs has become a topic of special concern, regardless of the type of solid organ (6–11).

The importance of an effective transition program for HTx has been universally recognized recently. The rate of midterm graft loss after HTx is higher in adolescents and young adults than in other age groups (9, 11). More than 50% of institutions in the US employ transition programs, while several pediatric centers continue to follow their patients even after they reach adulthood (11). The barriers to transition for HTx recipients include the requirement of regular inpatient and outpatient follow-up for rejection surveillance with trained transplant physicians possibly leading to unemployment and economic instability, and a sizable number of pediatric HTx recipients have other disabilities associated with congenital heart disease, such as developmental disorders (9). These issues will now be actively reviewed to explore optimal transition programs for HTx recipients worldwide.

Nevertheless, the pediatric-to-adult transition for HTx recipients has gained little attention in Japan to date. The first HTx surgery in Japan was performed in 1968, which was only 9 months after the first human HTx was carried out in South Africa by Bernard in 1967. However, the first case in Japan raised multiple concerns regarding the criminal liability of the surgical team, such as the misdiagnosis of brain death and inappropriate recipient selection (12), causing 30 subsequent years of domestic stagnation in transplant medicine. With the establishment of legal systems, “adult” HTx was re-carried out in 1999 under the Organ Transplant Law, but pediatric patients were still required to go abroad to undergo HTx surgery. The law was amended in 2010, and the first pediatric HTx for recipients younger than 6 years of age was performed in 2012. In recent years, the annual number of HTx surgeries performed in Japan has been about 50–70, including 3 to 17 pediatric cases. In total, 177 Japanese pediatric patients underwent HTx between 1988 and 2020, 124 of which were performed overseas (13). This is highly specific to Japanese pediatric HTx. The primary heart disease requiring HTx is mainly nonischemic cardiomyopathy, regardless of the age group in Japan, and the posttransplant survival rate is excellent, nearly 90% at 10 years (13). The post-HTx follow-up schedule is similar to other countries, requiring weekly visits during the early postoperative period, followed by monthly or bimonthly visits, together with protocol biopsies and surveillance for transplant vasculopathy. The pre- and post-HTx treatment fees are covered by the national health insurance system in Japan, although adult HTx recipients must pay up to 20%–30% of the cost depending on the type of insurance they hold and whether the primary heart disease is a designated intractable disease. For pediatric patients, the Medical Aid for Specific Chronic Pediatric Diseases pays the pre- and post-HTx medical fees until the recipient reaches 20 years old (14). However, as stated above, sizable Japanese pediatric recipients required overseas HTx until recently, of which the cost is out of pocket or fund-raised. Such tremendous costs are indeed a significant burden for parents. This unusual and tragic background of HTx in Japan delayed active discussion on any topic related to transplantation, including transition medicine.

## The road toward a heart transplant transition program in Japan

The Japan Pediatric Society officially announced that patients can be cared for by pediatricians until they reach 20 years old (15), and medical fees for pre- and post-HTx treatments are indeed covered by the government (14). Considering the recommendation from the American Academy of Family Physicians (AAFP) and American Academy of Pediatrics (AAP), which stated that the preparation for a transition plan should be started by age 14 (16), we propose that the actual transition program should be employed around age 15 and gradually adjusted toward the age of 20, according to the developmental stage and intellectual ability of each patient in Japan.

**Figure 1:**
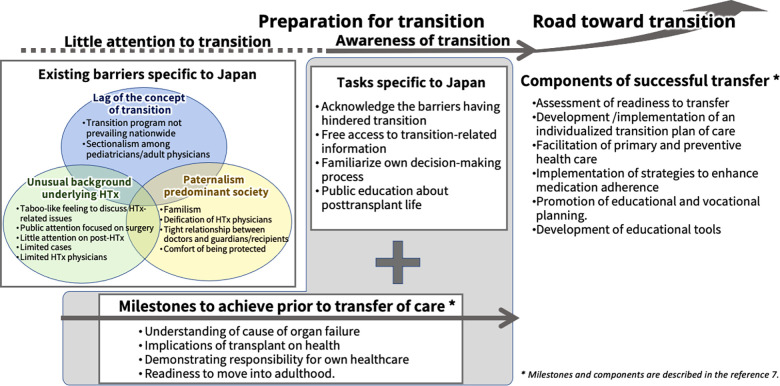
The conceptual framework for establishing a transition program for heart transplant recipients in Japan. The transition program has not been fully discussed to date in Japan, and the underlying reasons are described in the three balloons below. The roadmap to a successful transition starts with acknowledging the situation. Critical milestones to achieve and components of successful transfer for young adult patients after solid organ transplantation prior to transfer of care, as described in a consensus conference report (7), are shown in the figure.

Figure 1 describes several existing barriers to starting a discussion on the transition program for HTx recipients in Japan and a thinkable roadmap for a successful transition.

First, transition medicine itself in Japan is almost 20 years behind, e.g., the US and European countries (17). The term transition in healthcare was first introduced in Japan in 2006, and it has been only about 10 years since the Japanese Pediatric Society and the government-led committees conducted research or surveys on the healthcare transition in 2013. Since then, the concept of the healthcare transition has spread, and its importance has been recognized by healthcare providers; however, it has not yet reached the area of HTx. Studies related to the transition in Japan were initially driven by pediatric nephrologists (18) and mostly targeted neurology and nephrology, but few studies have included cardiology or solid organ transplantation. Both disease-specific studies and a questionnaire survey by Ishizaki et al. concluded that transition programs are necessary and should be expanded nationwide (19).

Second, the tragic episode of the first HTx in Japan, as described previously, led the Japanese population to begin considering the concept of HTx “special.” Central players, including physicians and recipients, are highly sensitive to any criticism against HTx and have established a strong relationship among each other. On the other hand, non-central players, such as the healthy general population and even healthcare professionals working in the non-transplant field, tend to consider it taboo to participate in discussions related to HTx. In such a society, undergoing HTx surgery itself is the goal, and the recipient's post-transplant life has not received much attention. Indeed, life-stage choices that pediatric HTx recipients will encounter, such as marriage, pregnancy, employment, and actual end-of-life care or re-transplant listing, have not been discussed fully to date in Japan. In addition, the number of cases is small, about one-seventieth of those in the US per capita annually (20). Therefore, it has been difficult to train both pediatric and adult HTx physicians or divide their roles.

Third, paternalism remains predominant in clinical settings in East Asia, including Japan (21), which can hinder the independent and active decision-making process of patients and their guardians. Until recently, it was unusual for Japanese, Korean, or Chinese doctors to convey the truth about a poor prognosis or treatment strategy directly to their patients (22, 23). In other words, patients were commonly left uninformed about their condition (22, 23). Several studies reported that this is related to Confucianism, whose philosophical background is so-called familism (22, 24). Consequently, medical paternalism tends to be a mainstream of clinical decision-making, especially in a process of treating pediatric cases. Besides, the historical background of HTx in Japan further encourages medical paternalism through the deification of transplant surgeons. It is quite understandable that not only recipients but also their guardians feel comfortable being seen by the same HTx physicians for extended periods, regardless of their age, especially when they have created strong relationships with their doctors. The recipients and their guardians feel protected through the behavior pattern of simply following the instructions of healthcare professionals.

Even having considered these barriers, pediatric HTx recipients must eventually stand on their own feet. They require a lifelong self-tailored regimen of post-transplant immunosuppressive drugs and infection prophylaxis, which is a part of recipients' lives on their own. We believe understanding the sociocultural aspects is the first step to starting a fruitful discussion of an effective transition for pediatric HTx recipients in Japan. The critical milestones to achieve and the components of a successful transition for young organ recipients before starting the actual transition are described in the Consensus Conference Report by Bell et al. (7). Irrespective of each society's specific sociocultural background, the milestones and components of successful transfer are universal. The key issues shown in the Consensus Conference Report are also included in Figure 1 (7).

## Steps for establishing an effective heart transplant transition program in Japan

A report shown in the Cochrane Review assessing the effectiveness of interventions to improve the transition of care revealed that they may be effective in transitional readiness but they led to a little difference in outcome (25). A randomized controlled trial that investigated the feasibility of a transition intervention for young HTx recipients showed it may be efficacious at 3 months, but no differences at 6 months for any outcomes, such as immunosuppressive levels, rejection, or mortality, were identified (26). Both studies were limited by their small sample size and short follow-up duration (25, 26). Still, we believe access to an optimal and effective transition program would vary among countries and firmly depend on their sociocultural background, including the insurance system. A disease-specific approach would be also required. Although the above-mentioned previous studies failed to prove the effectiveness of transition intervention, disease- and country-specific transition programs can produce a favorable effect on the outcome of the very patients being targeted. Therefore, we expect this paper to be the first step in discussing this issue to date for both patients and healthcare professionals in the area of HTx. Issues that hinder or delay discussion of the transition of pediatric HTx recipients to adult programs in Japan are multifactorial, as described above. Even so, it is time to discuss this topic in consideration of the increase in pediatric HTx recipients and the improvement of their survival into adulthood worldwide.

Possible first steps to begin the discussion are as follows: (i) HTx physicians and allied health professionals can recognize both the importance of transition and the risks associated with its process, (ii) HTx physicians and allied health professionals can acknowledge the barriers having hindered transition to date, (iii) HTx recipients and their guardians can freely access information on the transition from domestic and international viewpoints, (iv) HTx recipients and their guardians can recognize the importance of a gradual shift from parent- or physician-directed management to adolescent self-management, and (v) a specialized training program concerning transition for healthcare professionals can be established, including recipient coordinators, which may be the most important and effective step. In addition, (vi) public education to clarify that “HTx surgery itself” should not be the goal, but a joyful and independent post-transplant life, is necessary.

The above proposals seem specific to pediatric HTx in Japan, but the underlying ideas may be universal, and we believe such a discussion would help address the commonality among the issues that transition medicine is facing worldwide. In conclusion, the nurturing of transition programs for pediatric HTx in Japan is warranted. Pediatricians and adult HTx specialists should share these issues and start a discussion, which would promote HTx medicine and ensure better clinical outcomes among HTx recipients in Japan.
